# The Availability of Space Service for Inter-Satellite Links in Navigation Constellations

**DOI:** 10.3390/s16081327

**Published:** 2016-08-19

**Authors:** Yinyin Tang, Yueke Wang, Jianyun Chen

**Affiliations:** College of Mechatronics Engineering and Automation, National University of Defense Technology, Changsha 410073, China; tangyinyin@nudt.edu.cn (Y.T.); kdcjy@sina.com (J.C.)

**Keywords:** GNSS, inter-satellite link, space service volume, availability

## Abstract

Global navigation satellite systems (GNSS) are widely used in low Earth orbit (LEO) satellite navigation; however, their availability is poor for users in medium Earth orbits (MEO), and high Earth orbits (HEO). With the increasing demand for navigation from MEO and HEO users, the inadequate coverage of GNSS has emerged. Inter-satellite links (ISLs) are used for ranging and communication between navigation satellites and can also serve space users that are outside the navigation constellation. This paper aims to summarize their application method and analyze their service performance. The mathematical model of visibility is proposed and then the availability of time division ISLs is analyzed based on global grid points. The BeiDou navigation constellation is used as an example for numerical simulation. Simulation results show that the availability can be enhanced by scheduling more satellites and larger beams, while the presence of more users lowers the availability. The availability of navigation signals will be strengthened when combined with the signals from the ISLs. ISLs can improve the space service volume (SSV) of navigation constellations, and are therefore a promising method for navigation in MEO/HEO spacecraft.

## 1. Introduction

The Global Positioning System (GPS) was the first of several global navigation satellite systems (GNSS). Others include the GLONASS, GALILEO, and BeiDou systems. With the progress of science and technology, human activities are expanding from Earth into space, and even to deep space, so various types of spacecraft have emerged. Navigation through ground stations presents some drawbacks because they have insufficient coverage and poor geometric dilution of precision (GDOP) for high orbit users. However, space-based navigation systems such as GPS do not have these problems. GPS is used for precise orbit determination [1,2], while the feasibility of GPS when applied in geosynchronous orbit (GEO) is exploited [3], and the results of tests conducted on the GPS receiver called PiVoT (for positioning, velocity and timing) that is designed to operate in high Earth orbits (HEO) is presented [4]. The GPS service volume is divided into the terrestrial service volume (TSV) and the space service volume (SSV). The former begins at the surface of the Earth and extends to 3000 km altitude, while the latter consists of the medium Earth orbit (MEO) SSV extending from 3000 km to 8000 km in altitude, and the HEO/GEO SSV extending from 8000 km to 36,000 km in altitude [5]. Since the GNSS currently in use were developed for terrestrial-based users, their availability is excellent in the TSV. However, it is poor for users in MEO or HEO orbits, especially those orbits that are higher than the navigation satellites. Normal receivers need to be improved in order to adjust for the weak signals and this increases the difficulty of development.

Inter-satellite links (ISLs) are used to shorten the end-to-end delay in low Earth orbit (LEO) satellite communication system [6]. The flower constellations theory is applied to the design and optimization of constellations maximizing the global coverage and the network connectivity via ISLs [7], the use of ISLs in polar and near polar circular orbit constellation is examined [8], and the ISLs routing is discussed [9,10]. However, ISLs are used to provide ranging and allow communication between the navigation satellites. ISLs can also be used to improve the accuracy of navigation system for positioning, velocity and timing, achieve integrity monitoring and autonomous navigation, and increase flexibility and expansibility. ISLs working on ultra-high-frequency (UHF) band were first introduced to enhance service performance in GPS. Other GNSS, such as GALILEO and BeiDou, also plan to establish ISLs [11,12]. The next generation GPS will update its ISLs to Ka or V band (approximately 23 or 60 GHz) [13]. High gain directional antennas will be needed in order to overcome the propagation loss caused both by the longer propagation path and by the higher carrier frequency, scanning antennas will be required to address the relative motion of the navigation satellites, and time division multiplexing will be used to increase the flexibility of the ISLs. Therefore, we can schedule some or all of the ISLs to serve users outside the navigation constellation at specific times, which is referred to as the inter-satellite link extension application (ISLEA).

Current research is focused inside the navigation constellation. A new approach for determining the precise orbit and ephemeris of the GNSS using an ISL was developed [14], a method that changes non-coherent integration to coherent integration was proposed to increase the sensitivity of ISL signal acquisition [15], an approach for allocating power in inter-satellite ranging of the navigation constellation, which can degrade the total power consumption to 55.3%, was presented [16], etc. However, there are few studies on the ISLEA. The SSV of the GPS main lobe is given by the received power, availability, and pseudorange accuracy [5]. Similarly, both the main lobe and the side lobe of the BeiDou SSV are characterized in terms of the minimum received power, satellite visibility, pseudorange paccuracy, and geometric dilution of precision (GDOP) [17]. Therefore, the ISL SSV should also include these aspects. The received power is related to the length of the propagation path, and the pseudorange accuracy is determined by the clock error, ephemeris error, and received power [18]. The significant difference between the ISL and the navigation signal is the availability due to the time division spot beam. Thus, this paper focuses on the availability characteristics of the ISL SSV.

The next section describes the relative movements of the satellites in a navigation constellation. Section 3 analyzes the availability of the ISL signal. The performance of a time division ISL is simulated in Section 4. The final section presents our conclusions.

## 2. Relative Movement of Satellites in a Navigation Constellation

Most navigation constellations consist of MEO satellites located in orbit around 20,000 km. Because of the complex high-speed relative motion of the satellites, it is necessary to introduce the characteristics of the ISL signal before analyzing its availability. For example, the BeiDou constellation includes five GEO satellites, 27 MEO satellites, and three inclined geosynchronous orbit (IGSO) satellites. The altitude of the GEO satellites is 35,786 km, and they are located at $58.75^{\circ }\;E$, $80^{\circ }\;E$, $110.5^{\circ }\;E$, $140^{\circ }\;E$ and $160^{\circ }\;E$. The altitude of the MEO satellites is 21,528 km, the inclination is $55^{\circ }$, and they form a 24/3/1 walker constellation with three backup satellites. The altitude of the IGSO satellites is 35,786 km and the inclination is $55^{\circ }$. Using this information, the range and rangerate of two arbitrary satellites can be simulated using the Satellite Tool Kit (STK).

**Figure 1:**
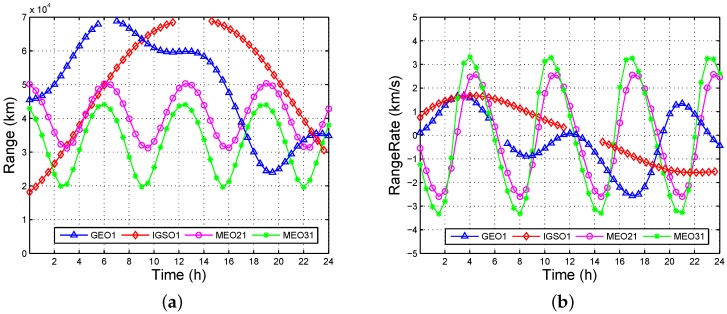
Characteristics of the ISL signal: (**a**) range to MEO11; and (**b**) rangerate to MEO11.

Figure 1 shows the line of sight (LOS) range and LOS rangerate from MEO11 to MEO21, MEO31, GEO1, and IGSO1, where MEOij is the *j*th (1,2,3,4,5,6,7,8) satellite in the *i*th (1,2,3) plane, GEO*m* is the *m*th (1,2,3,4,5) satellite from west to east, IGSO*n* is the *n*th (1,2,3) satellite with an initial phase increase. The gaps between GEO1 and IGSO1 are sheltered by the Earth. The range varies from 18,160 km to 68,847 km, and the rangerate varies from –3.33 km/s to 3.33 km/s. Hence, the effective SSV of the ISL is at least 68,847 km, which is the distance between the MEO to GEO. That is to say, the ISL signal can be directly used within the geosynchronous orbit, and achieve improved observation geometry.

## 3. Analysis of Availability

**Figure 2:**
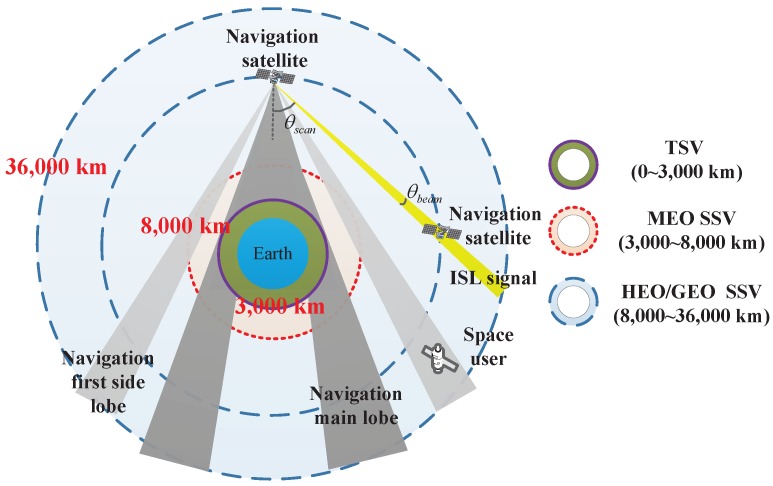
Coverage characteristics of navigation signal and ISL signal.

The ideal goal for navigation satellites is to ensure that all users in the service space can observe four or more satellites for real-time positioning, and the suboptimal goal is at least one satellite for time synchronization. The GPS service volume, which includes the TSV, MEO SSV, and HEO/GEO SSV, is shown in Figure 2. Numerical results reveal that 97% of the users in the MEO SSV can obtain four-fold availability using the main lobe of L1, while only 1% of the users can obtain four-fold availability, and 80% of the users can obtain single-fold availability at an altitude 36,000 km [5]. The availability may be enhanced if the side lobes are considered. However, there are two problems hidden in these seemingly satisfactory results: (1) the availability needs to be improved, especially in the HEO/GEO SSV; and (2) the navigation signal received at the GEO is approximately 25 dBW weaker than that at an altitude of 3000 km. Use of the ISL signal is expected to solve these problems because its natural advantage is that it is designed for users that are in space.

Figure 2 shows two additional parameters of the ISL signal: $\theta_{beam}$ is the beamwidth and is related to the gain of the antenna, and $\theta_{scan}$ is the scanning angle and is related to the field of view.

**Figure 3:**
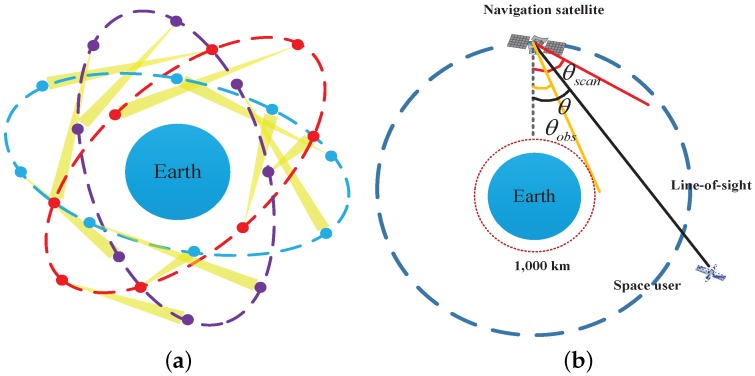
Geometry of ISLs at a specific time: (**a**) ISL signals among the navigation satellites; and (**b**) ISL signal to space users.

The availability of the ISL is defined as the number of visible navigation satellites at an arbitrary time. This assumes that the signal can be received as long as users are in the LOS of the antenna. In order to eliminate the influence of the ionosphere and troposphere, altitudes under 1000 km are considered to be invisible, as shown in Figure 3.

The off-axis angle *θ* is the angle between a line extending from the navigation satellite to the space user, and a line extending from the navigation satellite to the center of the earth. A navigation satellite that is visible to the space user must meet Equation (1):

(1) $$\left\{\begin{array}{c} \theta_{obs}<\theta <\theta_{scan},\;\;\;or\;\\ \;\theta_{obs}>\theta \;\;\;and\;\;\;\;∥\vec{R_{s}}-\vec{R_{u}}∥<\left(R_{E}+H_{s}\right)\cos\left(\theta_{obs}\right)\;\;\;\; \end{array}\right.$$

where $\theta_{obs}=\arcsin\left(\left(R_{E}+1000\right)/\left(R_{E}+H_{s}\right)\right)$ is the Earth obstructing angle, $\theta_{scan}$ is the maximum scanning angle, $R_{E}$ (6378.137 km) is the radius of the Earth, $H_{s}$ is the altitude of the navigation satellite, $\vec{R_{s}}=\left(x_{s},y_{s},z_{s}\right)$ is the position of the satellite in ECEF (Earth-Centered, Earth-Fixed) coordinates, and $\vec{R_{u}}=\left(x_{u},y_{u},z_{u}\right)$ is the position of the user in the same coordinate system. The upper condition means the user outside the maximum scanning angle is unavailable. The lower means the visible user and satellite should be in the same side of Earth if $\theta <\theta_{obs}$. The cosine of *θ* can be computed as

(2) $$\cos\left(\theta \right)=\frac{<\vec{R_{s}},\vec{R_{s}}-\vec{R_{u}}>}{∥\vec{R_{s}}∥∥\vec{R_{s}}-\vec{R_{u}}∥}$$

Hence, we get

(3) $$\theta =\arccos(\frac{x_{s}\left(x_{s}-x_{u}\right)+y_{s}\left(y_{s}-y_{u}\right)+z_{s}\left(z_{s}-z_{u}\right)}{\sqrt{{x_{s}}^{2}+{y_{s}}^{2}+{z_{s}}^{2}}\sqrt{{\left(x_{s}-x_{u}\right)}^{2}+{\left(y_{s}-y_{u}\right)}^{2}+{\left(z_{s}-z_{u}\right)}^{2}}})$$

The visibility can be determined by Equations (1) and (3).

## 4. Numerical Simulation

The number of navigation satellites that can be scheduled to serve for spacecraft is restricted by ISL’s own work and the time division spot beam limits the number of users that can be served simultaneously, although the availability may be enhanced if the navigation signal is combined. Therefore, this paper analyzes the availability in the following cases: (1) all of the satellites are scheduled to serve a single target (ASST); (2) only some of the satellites are scheduled to serve a single target (SSST); (3) all of the satellites are scheduled to serve multiple targets (ASMT); (4) only some of the satellites are scheduled to serve multiple targets (SSMT); (5) none of the satellites are scheduled (NS); and (6) the navigation signal is combined with the ISL (NSCI).

In this section, we present the results of the numerical simulation that we used to characterize the availability of ISLs. All of the simulations were implemented using M-files in MATLAB R2010a (MathWorks, Natick, MA, USA). The tested navigation system was the MEO satellites in BeiDou. The maximum scanning angle was $\pm 55^{\circ }$, the sphere was evenly divided into $6^{\circ }\times 6^{\circ }$ grids at each altitude, the step size was 60 s, the simulation time was 48 h. Table 1 lists the parameters and values used.

### 4.1. All of the Satellites Are Scheduled to Serve for a Single Target (ASST)

The availability of the ISL varies with the altitude and can be determined based on the statistics of each grid. The availability of an arbitrary grid point is the average of the visible satellites throughout the simulation time. The best location is the maximum availability of all the grids at an altitude, the worst location is the minimum availability at an altitude, and the global average location is the average availability at an altitude.

**Figure 4:**
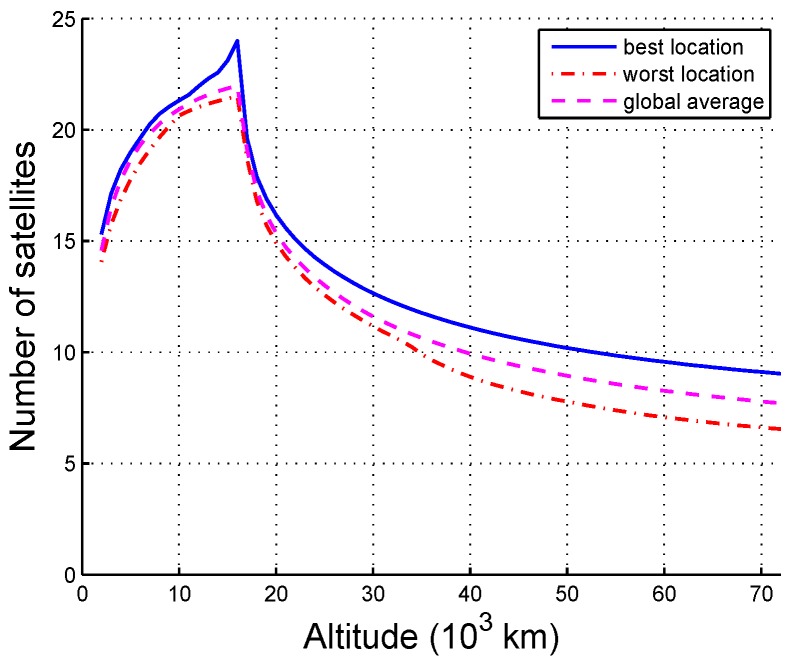
Availability as a function of altitude.

The availability of ASST is shown in Figure 4. Due to the impact of the Earth obstructing angle, the number of visible satellites increases at first and then decreases with altitude. The maximum number of visible satellites is 24 at an altitude of approximately 16,000 km, and the differences between the best location, the worst location, and the average location are rarely within 30,000 km, which reveals that the coverage of the ISL signals is uniform. Moreover, any location within 72,000 km reaches four-fold coverage. In other words, the navigation constellation SSV is significantly expanded by the ISLs, and this can effectively complement the inadequate coverage for the HEO/GEO SSV using the navigation signal. A prediction can be made based on the trends shown in the curves that the availability above 72,000 km will continue to drop resulting in the received power being too weak to acquire, although this is not the topic of this paper. Note that the ISL SSV is larger than the navigational SSV because of its wide scanning angle.

### 4.2. Only Some of the Satellites Are Scheduled to Serve for a Single Target (SSST)

In order to not affect the normal work of navigation constellations, only some of the satellites can be scheduled to serve other spacecraft. Assuming that the normal work can be maintained by the half of the satellites, three scenes consisting of 1/2, 1/4, 1/8 satellites were discussed: the satellites numbered 1, 3, 5, 7 of each orbit; the satellites numbered 1, 5 of each orbit; and the first satellite of each orbit, respectively.

**Figure 5:**
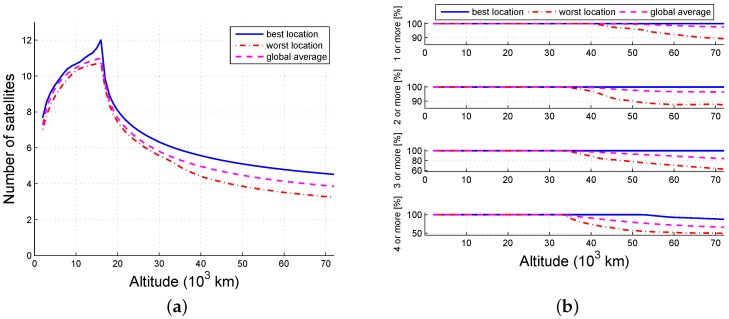
Availability as a function of altitude (1/2): (**a**) number and (**b**) probability.

**Figure 6:**
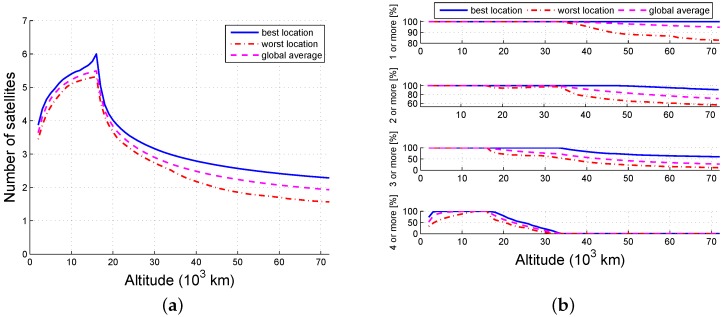
Availability as a function of altitude (1/4): (**a**) number and (**b**) probability.

**Figure 7:**
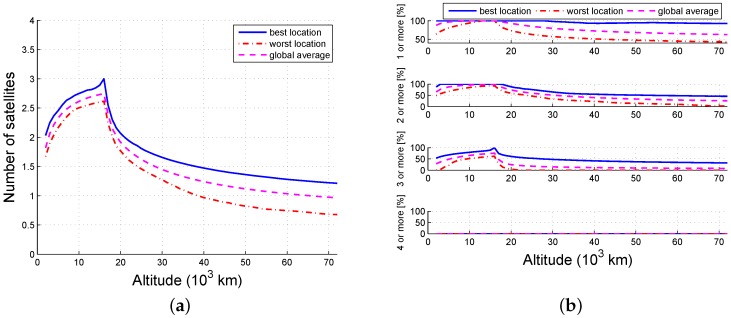
Availability as a function of altitude (1/8): (**a**) number and (**b**) probability.

The probability that an arbitrary grid point is available is the ratio of the visible time and the simulation time. Figure 5, Figure 6 and Figure 7 illustrate the tendency that part of the constellation is the same as the whole constellation. When there are 12 available satellites, all locations below 40,000 km and no less than 97.6% (global average) of the locations within 72,000 km can view at least one satellite; 100% of the locations lower than 34,000 km and no less than 96.2% (global average) of the locations within 36,000 km may view at least four satellites. When there are six available satellites, the number of locations that can view at least one satellite is 100% within 33,000 km and 95.1% (global average) within 70,000 km; the locations (global average) that can view at least four satellites are constrained to be at an altitude between 6000 km and 16,000 km, and no locations can reach four-fold coverage above 34,000 km. When the number of usable satellites is 3, then 95% of the locations between 4000 km and 18,000 km can get one-fold coverage. Thus, the availability of ISLs decreases as the number of scheduled satellite resources decreases.

Based on these results, the single-fold SSV can be extended to 70,000 km and the four-fold SSV can be expanded to 16,000 km when there are not many satellites scheduled (1/4).

### 4.3. All the Satellites Are Scheduled to Serve for Multiple Targets (ASMT)

**Figure 8:**
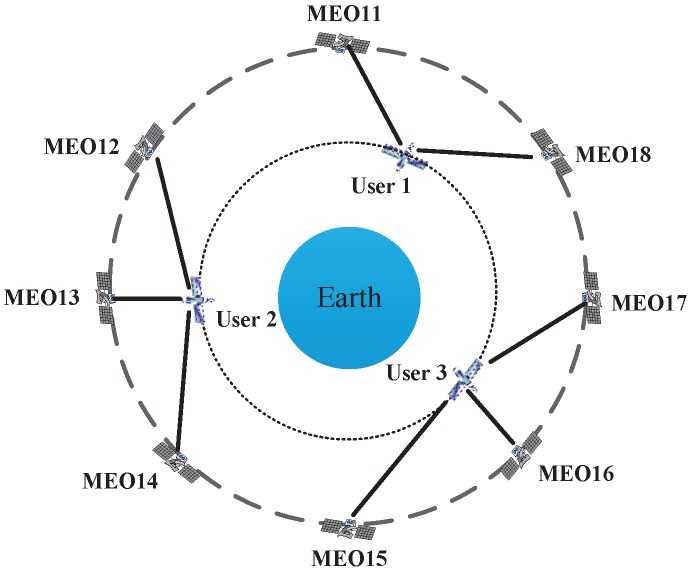
Satellite selection strategy of multiple users.

For simplicity, consider the case where multiple users are evenly located over the equator, as shown in Figure 8. In this case, the position of the *k*th user can be described as (Lon,Lat,*h*) in the WGS-84 reference system; where $Lon=360^{\circ }\;k/N,\;\;k=0,1,...,N-1$ denotes the longitude, Lat=0 denotes the latitude, *h* is the altitude, the main value is $Lon\in \left[-180^{\circ },\;180^{\circ }\right]$, and the user number $N\in \left[2,24\right]$.

The problem of determining which satellite will be assigned to which user is called the satellite selection strategy. In this paper, we used a minimum distance criterion that a satellite is prior to establishing an ISL with its nearest visible user. The distance between satellite *m* and user *n* is

(4) $$d_{mn}=\sqrt{{\left(x_{sm}-x_{un}\right)}^{2}+{\left(y_{sm}-y_{un}\right)}^{2}+{\left(z_{sm}-z_{un}\right)}^{2}}\;\;,m\in \left[1,24\right]\;,\;n\in \left[2,N\right]$$

Link(m,n) represents the link of satellite *m* and user *n*, thus $Link\left(m,n\right)=min\left(d_{mn}\right)$.

**Figure 9:**
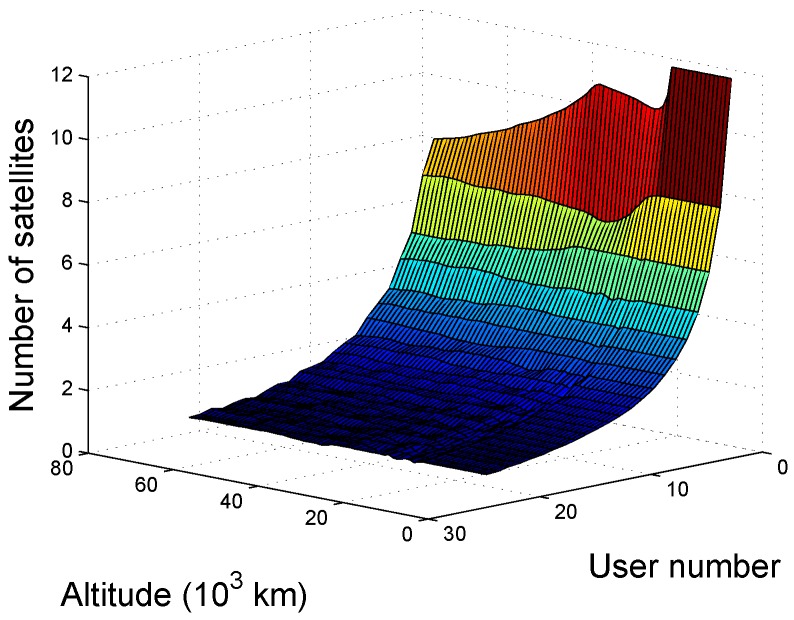
Availability of multiple users (the worst user).

The availability as a function of altitude and number of users is shown in Figure 9, where the average availability of the worst user during the simulation is based on the minimum distance criterion. As seen in the figure, the number of visible satellites is inversely proportional to the number of users because of the average function of multiple users.

**Figure 10:**
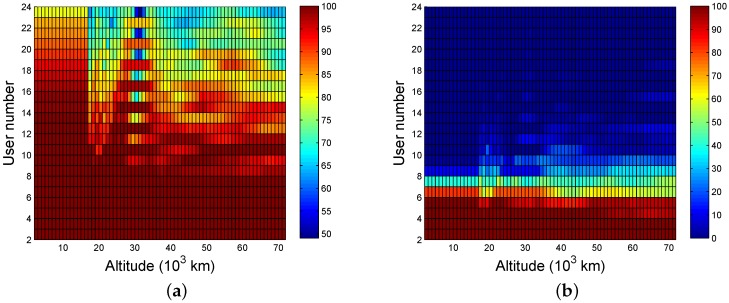
The available probability of ASMT (the worst user): (**a**) one or more; (**b**) four or more.

Figure 10a,b illustrate the probability of at least single-fold availability, and at least four-fold availability, respectively. In general, the availability can be considered strong if the probability is more than 95%. Strong single-fold availability can be achieved based on the conditions that there are fewer than 17 users and the altitudes are lower than 15,000 km, or there are fewer than eight users and the altitudes are lower than 72,000 km. The conditions to achieve four-fold coverage require less than four users or altitudes that are lower than 15,000 km if the users are five.

### 4.4. Only Some of the Satellites Are Scheduled to Serve for Multiple Targets (SSMT)

**Figure 11:**
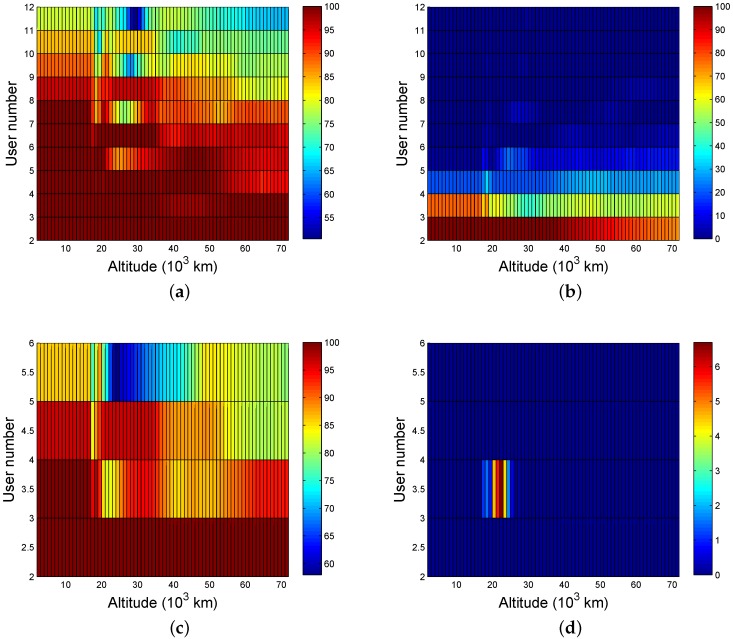
The available probability of SSMT (the worst user): (**a**) one or more (12 satellites); (**b**) four or more (12 satellites); (**c**) one or more (six satellites); and (**d**) four or more (six satellites).

Based on the analysis of the SSST and ASMT cases, we can easily determine the availability when either six or 12 satellites are scheduled, as shown in Figure 11.

The results in Figure 11 show that the availability decreases as the number of satellite resources decreases and the number of users increases. The number of users should be no more than eight for strong single-fold coverage when the number of scheduled satellites is 12, and the number of users should drop to three when the locations are higher than 50,000 km. In order to receive four-fold signals, the number of users must be limited to two and they must be within 40,000 km. Only single-fold coverage is possible when there are six satellites scheduled to serve users and, in order to achieve 95% availability, the number of users must be smaller than three or altitudes that are lower than 15,000 km if the users are four.

### 4.5. None of the Satellites Are Scheduled (NS)

**Figure 12:**
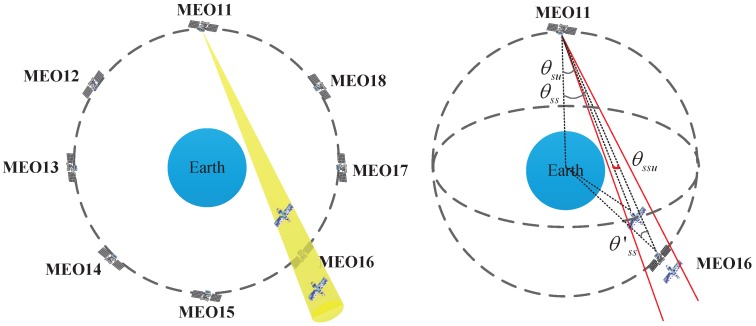
Geometry of users receive the signal by chance when ISLs are in normal work.

From time to time, there are essential and real-time activities using the ISLs that cannot be interrupted. Consequently, in these cases, the users only receive service sporadically, as shown in Figure 12.

In the NS case, the availability will be reduced significantly and is related to the beamwidth. Considering the visibility, users should be covered in the beam of the ISL and should not be obstructed by the Earth, that is:

(5) $$\left\{\begin{array}{c} \theta_{obs}<\theta_{ss}<\theta_{scan}\\ \theta_{obs}<\theta_{ss}^{\prime }<\theta_{scan}\\ \theta_{obs}<\theta_{su}\\ 0\leq \theta_{ssu}\leq \theta_{beam}/2 \end{array}\right.$$

where $\theta_{ss}$ is the angle formed by the LOS of the two navigation satellite and the line from the transmitting satellite to the center of the Earth , $\theta_{ss}^{\prime }$ is the angle formed by the LOS of the two navigation satellite and the line from the receiving satellite to the center of the Earth, $\theta_{su}$ is the angle formed by the LOS of the transmitting satellite to the user and the line from the transmitting satellite to the center of the Earth, and $\theta_{ssu}$ is the angle formed by the LOS of the two navigation satellite and the LOS of the transmitting satellite to the user. The first two conditions mean the two satellites can view each other so that a link can be established. The last two conditions guarantee the user is covered by the beam and is not obstructed by Earth. If the position of the transmitting satellite in ECEF coordinates is $\vec{R_{st}}=\left(x_{st},y_{st},z_{st}\right)$, the position of the receiving satellite is $\vec{R_{sr}}=\left(x_{sr},y_{sr},z_{sr}\right)$, the position of the user is $\vec{R_{u}}=\left(x_{u},y_{u},z_{u}\right)$, then the angles in Equation (5) can be computed as Equation (6) according to Equation (2):

(6) $$\left\{\begin{array}{c} \theta_{ss}=\arccos\left(\frac{x_{st}\left(x_{st}-x_{sr}\right)+y_{st}\left(y_{st}-y_{sr}\right)+z_{st}\left(z_{st}-z_{sr}\right)}{\sqrt{{x_{st}}^{2}+{y_{st}}^{2}+{z_{st}}^{2}}\sqrt{{\left(x_{st}-x_{sr}\right)}^{2}+{\left(y_{st}-y_{sr}\right)}^{2}+{\left(z_{st}-z_{sr}\right)}^{2}}}\right)\\ \theta_{ss}^{\prime }=\arccos\left(\frac{x_{sr}\left(x_{sr}-x_{st}\right)+y_{sr}\left(y_{sr}-y_{st}\right)+z_{sr}\left(z_{sr}-z_{st}\right)}{\sqrt{{x_{sr}}^{2}+{y_{sr}}^{2}+{z_{sr}}^{2}}\sqrt{{\left(x_{sr}-x_{st}\right)}^{2}+{\left(y_{sr}-y_{st}\right)}^{2}+{\left(z_{sr}-z_{st}\right)}^{2}}}\right)\\ \theta_{su}=\arccos\left(\frac{x_{st}\left(x_{st}-x_{su}\right)+y_{st}\left(y_{st}-y_{su}\right)+z_{st}\left(z_{st}-z_{su}\right)}{\sqrt{{x_{st}}^{2}+{y_{st}}^{2}+{z_{st}}^{2}}\sqrt{{\left(x_{st}-x_{su}\right)}^{2}+{\left(y_{st}-y_{su}\right)}^{2}+{\left(z_{st}-z_{su}\right)}^{2}}}\right)\\ \theta_{ssu}=\arccos\left(\frac{\left(x_{st}-x_{su}\right)\left(x_{st}-x_{sr}\right)+\left(y_{st}-y_{su}\right)\left(y_{st}-y_{sr}\right)+\left(z_{st}-z_{su}\right)\left(z_{st}-z_{sr}\right)}{\sqrt{{\left(x_{st}-x_{su}\right)}^{2}+{\left(y_{st}-y_{su}\right)}^{2}+{\left(z_{st}-z_{su}\right)}^{2}}\sqrt{{\left(x_{st}-x_{sr}\right)}^{2}+{\left(y_{st}-y_{sr}\right)}^{2}+{\left(z_{st}-z_{sr}\right)}^{2}}}\right) \end{array}\right.$$

The grid used in the simulation needs to be changed to $\theta_{beam}/2\times \theta_{beam}/2$ when the satellites are unscheduled. The timing and link are decided by the link plan, which is generated based on the visibilities and requirements. There are a maximum of 12 pairs of simultaneous links between 24 satellites. There are always four visible links in the same orbit and in the different orbits for each navigation satellite. Three kinds of link plans are analyzed in this paper: all links are in the same orbit, all links are in different orbits, and half are in the same orbit. The link plans are shown in Table 2, where 11–13 means the link of MEO11 and MEO13, etc.

**Figure 13:**
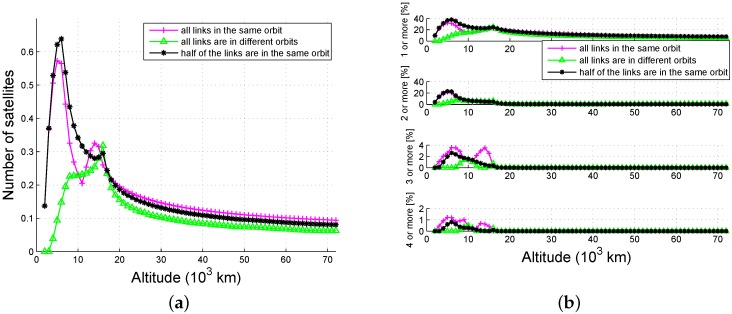
Availability as a function of link plan ($\theta_{beam}=12^{\circ }$): (**a**) number and (**b**) probability.

Figure 13 demonstrates the availability of global average as a function of the altitude and link plan when $\theta_{beam}=12^{\circ }$. From Figure 13a, we know that most of the satellites are seen when half of the links are in the same orbit, and the availability of links in the same orbit are much higher than those in different orbits. From Figure 13b, we can see that multiple-fold availability is nearly zero if the altitude is higher than 22,000 km, since only one of the two beams in a link can be received by users that are above the navigation satellites. The highest single-fold availability is approximately 40%, while the highest four-fold availability is only 1%. The availability as a function of beamwidth is simulated below.

**Figure 14:**
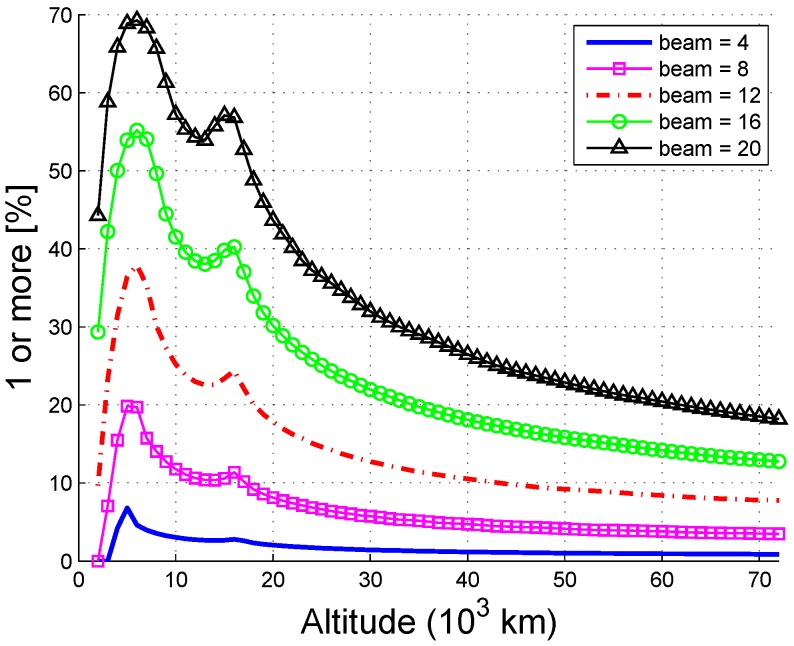
Availability as a function of beamwidth (single-fold).

Figure 14 shows that the availability increases with beamwidth, and there are two peaks at 6000 km and 14,000 km. The availability probability of users under 30,000 km may reach 30% or even to a maximum of 70% if the beamwidth is increased to $20^{\circ }$. Note that the beamwidth cannot be arbitrarily increased because this negatively affects the gain of the antenna. Therefore, both the availability and the received power should be considered when choosing the beamwidth.

### 4.6. Navigation Signal Combined with ISL (NSCI)

The availability of ISLs is far from ideal if the navigation satellites cannot be scheduled and the HEO/GEO SSV of the navigation signal needs to be enhanced. We next evaluate the availability when the ISL signals and the navigation signals are combined.

**Figure 15:**
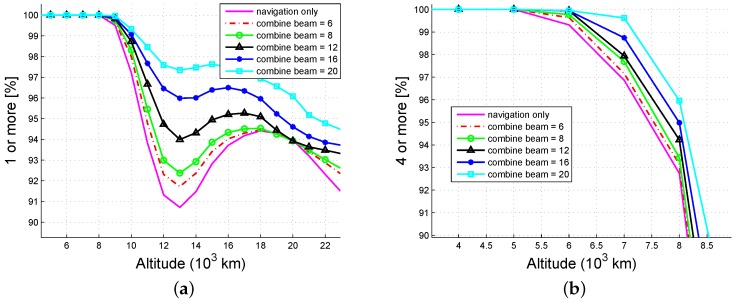
The combined availability of ISL and navigation signal: (**a**) one or more; (**b**) four or more.

Figure 15 illustrates the combined availability of the ISL signals and the navigation signals, where the main lobe of the navigation signal is $44^{\circ }$. The combined availability increases with the ISL beamwidth. When the beamwidth is $20^{\circ }$, the strong single-fold probability (≥95%) increases from 10,000 km to 21,000 km (an increase of 110%), the strong four-fold probability (≥95%) is extended from 7400 km to 8200 km, and the increased altitude is 800 km.

## 5. Conclusions

This paper presents the derivation and numerical results for ISL availability. The performance of availability is shown to be related to the altitude, resources scheduled, number of users, beamwidth, and whether the ISL signals are kept separate or combined with other signals.

The availability decreases with smaller scheduling satellite. The four-fold coverage expands to 72,000 km if all satellites can be scheduled to serve for space users, and the performance is satisfactory even when the number of scheduled satellites is six (single-fold to 70,000 km, and four-fold to 16,000 km). The availability decreases with more users. The maximum number of simultaneous users served by ISLs is 16 for single-fold availability but is only three when the requirements change to four-fold availability. The availability increases with larger beamwidth. The availability is poor if no satellites can be scheduled, since single-fold reaches only 6.8% when the beamwidth is $4^{\circ }$, yet the probability increases to 70% when the beamwidth is $20^{\circ }$. Although the ISL availability is not satisfactory in this case, it can be used to enhance the availability of the navigation signal, as the single-fold SSV is increased 110% and the four-fold SSV is extended 800 km when the beamwidth is $20^{\circ }$.

In the near future, ISLs will be widely applied in all aspects of space services and will become another fundamental resource for navigation satellites. In order to further enhance the availability of ISLs, we plan to consider incorporating the side lobe in our analysis, along with other GNSS.

## Figures and Tables

**Table 1:** Parameters used in the numerical simulation.

Parameter	Value	Comment
Grid Points	1800	$6^{\circ }\times 6^{\circ }$ grids
Duration	48 h	Length of simulation time
Step size	1 min	Computed every minute
Scanning angle	$\pm 55^{\circ }$	Maximum angle of the antenna
Beamwidth	$6^{\circ }$	Width of the spot beam
Altitude	(2000–72,000) km	Within twice the geosynchronous orbit
BeiDou-MEO	24	24/3/1 walker constellation
Visible constraint	Geometry	Line of sight except Earth obstructing
Start epoch	March 2016	Simulation epoch is arbitrary

**Table 2:** Link plans used in the numerical simulation.

In the Same Orbit	In Different Orbits	Half in the Same Orbit
11-13, 12-15, 14-17, 16-18	11-21, 13-23, 15-25, 17-27	11-21, 15-25, 13-18, 14-17
21-23, 22-25, 24-27, 26-28	22-32, 24-34, 26-36, 28-38	22-32, 26-36, 23-28, 24-27
31-33, 32-35, 34-37, 36-38	12-31, 14-33, 16-35, 18-37	12-31, 16-35, 33-38, 34-37
